# A Low Cost Concept for Data Acquisition Systems Applied to Decentralized Renewable Energy Plants

**DOI:** 10.3390/s110100743

**Published:** 2011-01-12

**Authors:** Sandro C. S. Jucá, Paulo C. M. Carvalho, Fábio T. Brito

**Affiliations:** 1 Federal Institute of Education, Science and Technology of Ceará—IFCE/Av. Contorno Norte, 10, Maracanaú, CE 61925-315, Brazil; E-Mail: fabio@ifce.edu.br; 2 Department of Electrical Engineering, Campus do Pici, Federal University of Ceará—UFC, Fortaleza, CE 60455-760, Brazil; E-Mail: carvalho@dee.ufc.br

**Keywords:** monitoring, data acquisition systems, renewable energy

## Abstract

The present paper describes experiences of the use of monitoring and data acquisition systems (DAS) and proposes a new concept of a low cost DAS applied to decentralized renewable energy (RE) plants with an USB interface. The use of such systems contributes to disseminate these plants, recognizing in real time local energy resources, monitoring energy conversion efficiency and sending information concerning failures. These aspects are important, mainly for developing countries, where decentralized power plants based on renewable sources are in some cases the best option for supplying electricity to rural areas. Nevertheless, the cost of commercial DAS is still a barrier for a greater dissemination of such systems in developing countries. The proposed USB based DAS presents a new dual clock operation philosophy, in which the acquisition system contains two clock sources for parallel information processing from different communication protocols. To ensure the low cost of the DAS and to promote the dissemination of this technology in developing countries, the proposed data acquisition firmware and the software for USB microcontrollers programming is a free and open source software, executable in the Linux and Windows® operating systems.

## 1. Introduction

Most energy projections show that current and expected future global energy demand patterns are not sustainable. Long-term projections indicate that the world energy demand may increase dramatically, with most of this increase taking place in developing countries. For instance, according to projections made by the World Bank and the International Monetary Fund, Asia and South America are believed to present higher growth rates than the rest of the world within the next decade [1]. The population of these regions must continue to grow faster than other countries and at the same time, there has to be an improvement of economic conditions, whereby creating a greater demand for more household appliances, industries and services, and hence, an increase in energy consumption. Today, at an overall world level, RE sources account for 13% of the primary energy demand (80% comes from fossil fuels, 7% from nuclear power), 18% of the electricity generation and 26% of the heat supply [2]. In developing countries RE plants, characterized by a low impact on the environment, can play a greater role in order to achieve low-carbon intensive energy systems to supply the increasing energy demand.

Decentralized electric energy generation, whereby generators are placed closer to consumption areas in order to overcome transmission loss which inherently increases with cable length, is a sustainable alternative. When this kind of power generation uses RE sources, there is a possibility to make use of local resources and even increase employment and income. Systems that are less centralized and less capital intensive may produce more job opportunities, thus the equation of available labor with the specific needs of each nation assumes great importance as erroneous planning could generate large scale social problems, especially in developing countries whereby there is a requirement for a large number of new jobs at the lowest possible level of investment [1]. Decentralization should be stressed to help promote a fair and desirable level of technological, economic and social development in these countries.

Monitoring and data acquisition are essential to recognize local RE resources, to monitor energy conversion efficiency and to send failure reports using intercommunication systems. The cost of wind energy converters and photovoltaic modules are still high in developing countries, whereby there is a requirement for a precise characterization of the energy resources and energy demand to find an optimal financial and technical alternative. On the other hand, DAS is an imported technology for most developing countries, meaning high cost and a barrier to the dissemination of such systems. The design and implementation of a low cost DAS is motivated by the need to offer an alternative for commercial systems, which are more expensive, usually imported and have proprietary software. In additional to this, commercial DAS do not allow amendments and adjustments to hardware nor software.

## 2. Literature Review of DAS Applied to RE Plants

The rapid development of the use of RE over the last decades has resulted in many plants being installed throughout the world. However, this effort requires, as a first step, a detailed knowledge of the meteorological data of the plant site; as a second step, operational values from similar RE plants are necessary to increase the technology reliability.

Balan *et al*. [3] presented a microcontroller-based DAS in which values of total and diffuse solar radiation intensities are captured by a microcontroller, using an electronic conversion module. The transmission of recorded values to a PC is carried out using a RS-232 serial interface. The acquisition software operates with a time step of 50 seconds, thereby reading and storing into a database the total and diffused solar radiation values. The database (MySQL type) is designed to store the values of measured solar radiation intensities and allows their interrogation via internet. In this manner, the PC also acts as a server computer for distance monitoring.

Belmili *et al.* [4] presented a microcontroller-based DAS for the characterization of PV modules under real meteorological test conditions. The DAS communication channel is a TTL/RS-232 transceiver that permits the serial interfacing between a microcontroller with Transistor-Transistor Logic (TTL) voltage levels and a computer (RS-232 voltage levels). The basic element of the DAS hardware is an electronic load based on MOSFET. The microcontroller is the central element of the designed DAS card, executing the electronic load variation, the analog-digital conversion from the solar irradiance, the communication with the 1-Wire temperature sensor and the data transmission to and from the computer via RS-232 interface. By using different buttons on a computer graphical interface, it is possible to visualize the results of the module characterization.

A radio frequency (RF) wireless DAS was developed in [5] with a set of sensors for measuring meteorological parameters (solar radiation, wind direction and speed, temperature, humidity and barometric pressure). The wireless DAS consists of two stations: a remote station (with sensors, microcontroller, RF emitter circuit and power supply) and a base station (with a RF receiver circuit, microcontroller, RS-232 serial interface and a computer with graphical environment software for processing, displaying and storing the collected data). A DAS which communicates with a server computer by means of an RF transceiver is also used in [6].

In [7] and [8] a microcontroller-based DAS is used in a remote PV plant. The measurement system uses a silicon-cell pyranometer as a solar radiation sensor. The sensor data is collected by an internal A/D converter of the microcontroller and stored in a serial I^2^C EEPROM until uploaded to a portable computer. Keeping the DAS in a low-power mode, which is only interrupted when measurements are to be taken or when a computer is connected to retrieve the stored data, it is possible to minimize the power consumption. An on-chip timer provides a way to change operational conditions, whereby changing the low-power wait mode at 10-min intervals to sample and store the data. At the end of each data collection period, the acquired data is transmitted to the computer through a RS-232 serial interface.

In [9] a DAS applied to a PV plant that is capable of delivering 5 kWp of electrical power was described. Temperature, irradiance, array voltage and current data are acquired, processed and then transmitted. The system comprises sensors, DAS, wireless access point and computer, enabling users to access the array parameter via a wireless connection. Three current sensors with a maximum current value of 50 A and a sensitivity of 37.8 mV/A, are connected to the dc array output, while irradiance and temperature sensors are placed around the array. For voltage acquisition, a voltage divider was used, which means 10 mV for every one volt of solar panel output. LM35 temperature sensor with sensitivity of 10 mV/°C is used to measure the ambient temperature of the solar panel. The irradiance is sensed using LDR. The microcontroller serial interface is connected to an EGSR7150 modem in order to convert format data from serial to Ethernet data. Furthermore, the acquired data is also sent to the access point of the connected user computer in the form of a Wi-Fi signal (IEEE 802.11 standard).

A data transmission technology of ample expansion in the area of telemetry for remote regions is the GSM (Global System Mobile Communications) modem, using GPRS (General Package Radio Service). Currently, this technology is the most popular standard for mobile telephones around the world. GPRS application is a net connection for data package transmission. During a net connection, whereby the system is online, data is transfered immediately and the tariff is only made on the volume of data transmitted without considering the connection time. As GPRS is compatible with the TCP/IP protocol, GSM operators supply a gateway to the Internet, making it possible to connect and to control wireless equipment in this way. In [10], the sensor information is routed to the microcontroller, where it is processed and later sent to an external EEPROM memory and subsequently passed to the GSM/GPRS every 24 hours by RS-232 interface. In the case of a data transmission failure, caused by a hardware or a GSM modem problem, data remains stored in the memory.

An integrated DAS for RE sources systems monitoring is developed in [11]. A set of sensors are used to measure atmospheric and soil conditions, as well as quantities regarding the electricity produced by a hybrid PV/wind generator power system, such as PV array voltage and current, the wind generator speed. The collected data are further processed, displayed on the monitor and stored in hard disk.

In [12] voltage signals are connected together with a 2.5 V reference voltage to an external serial 12 bit A/D converter. The DAS microcontroller controls the electrical power-up and conversion start of the A/D converter by means of an internal serial communication link. The acquired values are then stored in an EEPROM (64 Mbits) for later retrieval, whereby the sensors are connected to a personal computer by means of a standard RS-232 serial transceiver.

The first large-scale application of DAS in PV powered water pumping plants in developing countries is the Mobile Data Acquisition System (MODAS 1220) created in 1991. Table 1 shows the main characteristics of the mentioned DAS.

The MODAS system has 16 AD converter of 12-bit resolution for analog input, data storage of up to 130,000 values in a CMOS-RAM and RS-232 serial interface for transmitting data to a PC. The GTZ (*Deutsche Gesellschaft Für Technische Zusammenarbeit*) implemented in the 1990s, the MODAS 1220 into the “International Program for Field Testing of Photovoltaic Water Pumps (PVP Programs)” in cooperation with the national energy and water authorities of Brazil, Argentina, Indonesia, Jordan, the Philippines, Tunisia and Zimbabwe. In the course of the PVP Programs, a total of 90 PVP systems were installed at selected sites in the project countries. Those systems provide potable water to rural communities and their livestock. It has been shown that PV pumping systems had a cost advantage over diesel pumping systems in the power range of up to 4 KW of electrical power [13].

## 3. Proposal of a Low Cost Concept for DAS Applied to Decentralized RE Plants

This section describes a low cost concept for a DAS applied to decentralized RE plants for use in developing countries, based on free software and an USB interface. Considering that most of DAS found in literature use a serial TTL/RS-232 transceiver for the connection to a PC, the proposed DAS represents a new use of low cost USB based systems. The motivation is that currently USB represents the most diffused peripheral-to-PC connection standard thanks to its flexibility, expandability and ease of use. In this way, there are two possibilities for data collection: data can be locally collected with the USB channel or remotely by GPRS, aiming to be stored on a data base, making possible a posterior analysis.

In most of the mentioned references, the data storage is done on a PC hard disk, whereby there is a requirement to provide constantly, a dedicated powered PC on-site only for this purpose, which makes the monitoring system more expensive. In the proposed DAS, the variables are stored directly in the DAS EEPROM, making the complete monitoring system cheaper. Although DAS EEPROM has a smaller capacity than the PC hard disk, other EEPROM can also be placed in parallel in order to increase the storage capacity.

Equipments designed and built for specific applications tend to be less costly, enhance performance and to provide user-friendly environment for control and communication [14]. Thus, the components price for the developed DAS is around € 50. This figure can be compared with the usual price in Brazil of commercial data loggers applied to RE sources of around € 2,000. It is clear that a commercial data logger has a greater operation range, but for specific applications, it makes sense to use a cheaper developed version.

The used microcontroller with USB interface manages all the I^2^C chips and the USB connection with the PC. The USB channel is used by a computer for setting the RTC RAM and data readings from the external EEPROM, through virtual serial channel emulation. Additionally, the USB channel is used for programming and updating the microcontroller firmware. The proposed low cost DAS is characterized by having a general application for analog and digital data and hardware simplicity, allowing also, data storage during long periods of time (months) without human intervention [15].

**Figure 1.**
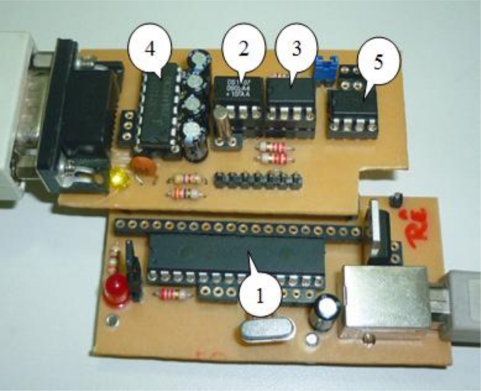
Developed low cost DAS card.

According to Figure 1, the developed USB based DAS is composed basically of an internal USB interface microcontroller (1), a real time clock (RTC) (2) and an external EEPROM (3), which communicates using an I^2^C twin wire bidirectional communication interface (I^2^C means Inter-Integrated Circuit or communication between integrated circuits). The acquisition system also has a hardware communication interface in the TTL/RS-232 transceiver (4), which can be used as interface in GSM/GPRS modems [16,17] and a TTL/RS-485 transceiver (5) for use in Modbus devices networks.

**Figure 2.**
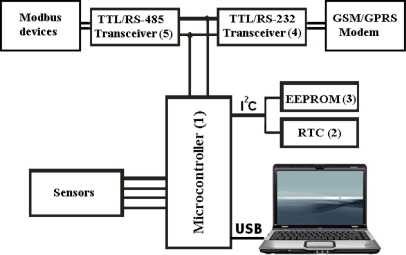
Simplified diagram of the proposed DAS.

Storage data can be read locally via USB interface or can be transmitted by wireless to servers through GSM/GPRS modem or to a Modbus network using the TTL/RS-485 transceiver. Figure 2 shows a simplified diagram of the proposed DAS.

In the microcontroller firmware, the sensors data is stored at predetermined time intervals in the external EEPROM via the I^2^C bus. Every second, the microcontroller performs a reading of the RTC via the I^2^C bus with the internal timer interrupt, checking the date (day, month and year) and time (second, minute and hour). If the RTC reading time is equal to the time set by the programmer to store data, the microcontroller writes the indicated sensors value in the external EEPROM memory.

The data storage in the PC is carried out by free monitoring software via USB emulation of a virtual serial channel. By using the same software, it is possible to set up and record the memories of the monitoring and acquisition system, such as the microcontroller memories, the RTC RAM and the external EEPROM, where the sensors variables are stored.

In addition, the microcontroller program memory can be written simply and directly via the USB interface and also through the developed *SanUSB* tool [18]. Thus, the system features can be altered through changes in the microcontroller program, for example, changing the frequency at which data is stored in the external EEPROM or changing the inputs configuration of analog-digital converters. Due to the diffusion of GSM devices, the transmission system is fairly cheap and it is expected to become even cheaper [19]. Therefore, this GSM modem system can be remotely monitored through a mobile network, with the possibility of displaying all of the acquired data to remote users in real-time through the Internet [20].

The *SanUSB* tool is the acquisition hardware core and is composed of software and a circuit based on the microcontroller with USB interface for the development of embedded systems [21], *i.e*., systems that manipulate data inside another larger system [22], as is the case of the proposed DAS.

To ensure the DAS low cost and to facilitate the tool dissemination, the software for the USB firmware microcontrollers and for the PC monitoring interface is free and open source, executable in Linux and Windows® operating systems and available from the archives at http://www.tinyurl.com/ SanUSB [18]. Using this tool, students were consecutively three time champions of the IFCE Robotics Competition (2007, 2008 and 2009), champions of the Brazilian Fair of Science and Engineering (FEBRACE09) in the University of São Paulo in Engineering Category (2009), and obtained the Innovation Award for Technology Application at the 2009 *Feria Explora* Medellin in Colombia and became Champions in the International Forum of Science and Engineering 2010 in Chile in the *Supranivel* Category [23].

Free and open source software refers to software that is distributed with a source code, often under a license that sets conditions for modification, reuse and re-distribution. Free and open source software is based on voluntary contributions over the Internet and from a community comprising geographically distributed developers. This software often offers the more reliable alternative, performs better, encourages creativity and can find and fix defects more rapidly [24], compared to proprietary software. Additionally, the software is globally available and growing in programmer participation and market share. The most recognized free and open source software practitioners reside in developed countries, but support from developing countries has surged in the last few years [25]. Using the *SanUSB* tool, it is possible to suppress the following items in the development of embedded systems:

- A specific device for microcontroller memory programming;
- TTL/RS-232 interface for bi-directional serial communication protocol emulated by USB communication device class (CDC);
- Power supply, since the power source is derived from the PC USB channel;
- Externally analog-digital converter (ADC), because the microcontroller has internally 10 ADC of 10 bits. Using externally ADC increases the cost of the data acquisition card [26];
- Simulation software, whereas the simulation program and hardware can be made quickly and effectively in their own development circuit.

Besides all these advantages, modern laptops and computers of today no longer have parallel and serial EIA/RS-232 communication interfaces, only USB. In this case, in order to read the acquired data or to set up the DAS at the plant site with a mobile computer, a system with USB interface is therefore recommended. The *SanUSB* tool enables user-friendly programming and real debugging directly through the internal microcontroller USB interface, as well as the serial communication through the virtual serial emulation. This can be created quickly and effectively at the very moment the microcontroller is connected directly to a PC, through the USB interface.

The I^2^C bus is designed with the goal of connecting integrated circuits and peripheral devices from different manufacturers on the same circuit using two pins. Microcontrollers, memories and external RTC can be included within the context of I^2^C devices. The I^2^C communication protocol is widespread and is usually implemented within hardware devices. For greater control of the connections between the system devices, the present project uses a protocol implementation via software.

**Figure 3.**
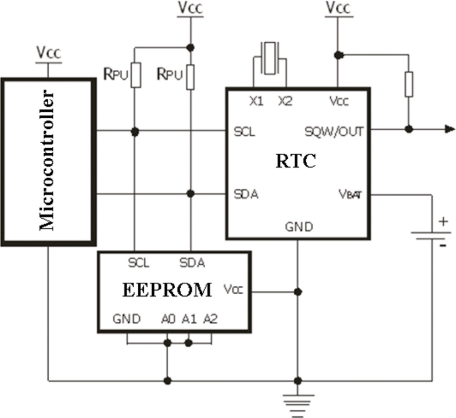
Connection scheme between devices using the I^2^C interface.

Most of microcontroller models have internal EEPROM, with a memory capacity of 256 bytes. The internal EEPROM is adequate for storing initialization parameters or retaining measured values during an operational sensing. Thus, a critical factor associated with data acquisition, is the microcontroller internal memory capacity. To overcome this limitation, an external EEPROM memory is included in the present system [27]. In the developed DAS, an option was made for an EEPROM model which provides 32 Kbytes with two months storage capacity and a RTC that is a low cost clock/calendar referenced by an external crystal at a frequency of 32.768 Hz. The connection Scheme between devices using the I^2^C interface in the proposed DAS is shown in Figure 3.

Aiming to manipulate and to check the I^2^C devices status, an ASCII serial protocol was implemented for communication between free monitoring software and DAS card through a virtual serial channel emulated by USB interface.

The microcontroller used with the USB interface has 21 pins available for digital sensors and up to 10 of these pins can be configured for analog sensors with a scale of 0 to 5 V, having as an advantage, the status of being a microcontroller widespread in the world.

This microcontroller also contains 32 Kbytes of program memory and a 21 bit program counter. Thus, when working with programs in C language, containing several applications within the main function or the timer interrupts function, as is the case of the DAS, it is recommended that a microcontroller with this capability is used.

It is important to note that the power supply from the USB is only used by the microcontroller during the logic programming development. Due to the autonomy, DAS can be installed using an external power supply.

**Figure 4.**
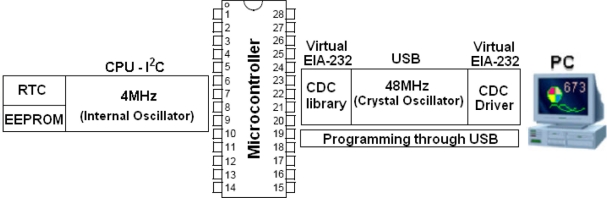
Developed dual clock system.

Due to incompatible frequencies between the USB and I^2^C interface, an USB dual clock system was developed using two clock sources. The first generates a clock source of 48 MHz for the USB channel, whereby an original frequency of 20 MHz from the external crystal oscillator is multiplied by an internal prescaler. The second clock source of 4 MHz is derived from the internal resistor-capacitor oscillator, as illustrated in Figure 4, for use in the internal microcontroller processor and for the I^2^C protocol.

This embedded system allows the users to insert data configurations and data readings using a computer keyboard or monitoring software. Data flows to the microcontroller at 48 MHz via the USB interface then directed to the RTC or external memory at 4 MHz via I^2^C. In this new concept, which combines parallel I^2^C protocol with the emulation serial via USB, the user can check the configuration with data returning from devices, through the same route.

## 4. Applications of the Low Cost DAS Applied to Decentralized RE Plants

**Figure 5.**
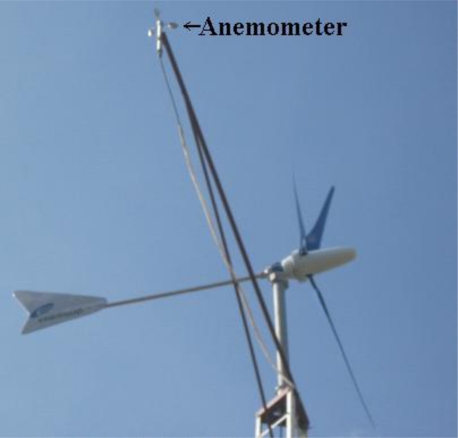
Anemometer at the UFC Campus, Fortaleza, Brazil.

The developed DAS has a generic application, being easily configured as digital or analog and having the possibility of being reprogrammed via the USB interface. In order to verify the applications for analog and digital sensors, the developed DAS was used to measure digital wind speed data from an anemometer (Figure 5) at a height of 10 meters and analog pressure and voltage data from a PV powered water pumping unity.

**Figure 6.**
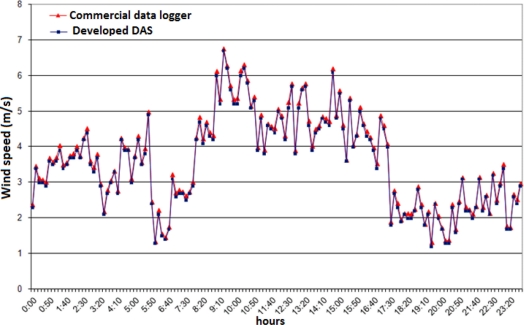
Wind speed data collected by the developed DAS and compared with the same data collected by a commercial data logger.

Figure 6 shows wind speed data collected by the developed DAS and compared with the same data collected by a commercial data logger (period: October, 29^st^ 2010).

**Figure 7.**
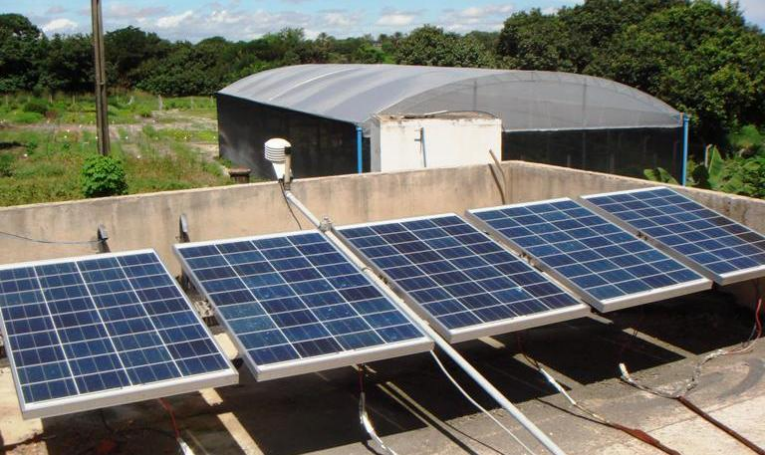
PV modules at the UFC Campus, Fortaleza, Brazil.

Analog pressure and voltage data came from a PV powered dc motor-pump used in a reverse osmosis desalination plant without batteries [28]. The PV array has five modules (Figure 7) with an individual power rating of 87 Wp.

**Figure 8.**
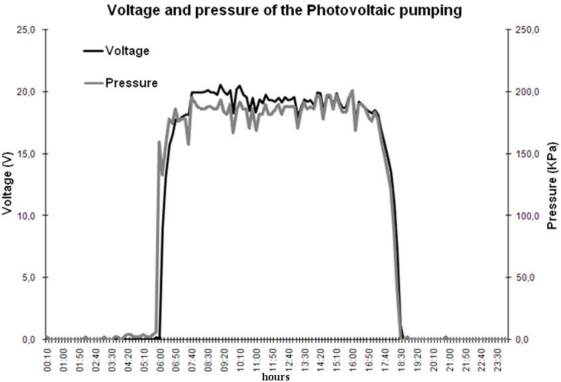
Voltage and pressure graph of a PV powered dc motor-pump.

The developed DAS was configured to perform a voltage and pressure reading every minute and store the average of this data in the external EEPROM every 10 minutes. From this data, a voltage and pressure graph is shown in Figure 8 (21 April 2010).

The implemented pressure sensor provides a voltage signal proportional to pressure. This sensor gives an analog signal of 0–10 V, equivalent to a pressure of 0–140 psi. Thus, for a maximum analog-digital converter voltage of 5 V, the pressure sensor indicates a maximal value of 70 psi (482.65 kPa). As shown in Figure 8, the motor-pump pressure follows the PV array voltage (for the best irradiation values, pressure is around 200 kPa and voltage is ca. 20 V). In this way, the developed DAS shows significant results in the digital application with a wind variable and in the analog application with PV voltage and pressure variables.

## 5. Conclusions

The use of low cost DAS will facilitate the spread of the measurements in RE plants, thus allowing recognized local energy resources such as solar radiation and wind speed to monitor the power conversion efficiency and to see possible failures in real-time for immediate correction.

Since the financial resources of developing countries are limited, low cost solutions are welcome to contribute to a decentralized power generation policy. In the developed DAS, an USB channel, the most diffused peripheral-to-PC connections standard, was used to program the firmware microcontroller and to emulate a virtual serial communication with the PC.

The proposed USB based DAS was implemented to acquire wind speed information from an anemometer and voltage and pressure values of a PV powered motor-pump without batteries, demonstrating an acceptable and precise measurement of data. Among the main features is the use of an USB interface for data reading from the acquisition hardware, microcontroller programming and I^2^C peripherals configuration with the possibility of using standard serial communication via EIA/RS-485 for applications in MODBUS networks, in addition to the standard communication EIA/RS-232, which can be used as an interface for GSM/GPRS modules for data transmission in wireless networks.

The set up and writing of the RTC, the internal EEPROM and external EEPROM were evaluated using simple commands via the virtual serial interface by USB emulation. In addition, the microcontroller program memory showed the ease of writing in a simple and direct manner by using the *SanUSB* tool. Another advantage of the developed DAS, besides the efficiency and reliability, is the financial factor, due to the use of low cost and easy availability of components in the world market.

The use of supervisory systems and databases with free software provide a number of advantages compared to proprietary software, thereby reducing the system cost and ensuring total flexibility with the use of open source protocols, such as MODBUS. Finally, the proposed DAS works successfully and gives good performance, sensitivity, reliability and easy programming in both Linux and Windows^®^ operating systems.

## Figures and Tables

**Table 1.** Main Characteristics of the Mentioned DAS.

**Authors**	**Connection to the PC**	**Permanent data storage**	**Internet access**
Balan *et al.*, 2008 [3]	TTL/RS-232 transceiver	PC hard disk	yes
Belmili *et al.*, 2010 [4]	TTL/RS-232 transceiver	PC hard disk	no
Benghanem, 2010 [5]	RF transceiver	PC hard disk	yes
Kalaitzakis *et al.*, 2003 [6]	RF transceiver	PC hard disk	yes
Mukaro and Carelse, 2008 [7]	TTL/RS-232 transceiver	External EEPROM	no
Mukaro *et al.*,1998 [8]	TTL/RS-232 transceiver	External EEPROM	no
Anwari *et al*., 2009 [9]	Serial to ethernet module	PC hard disk	yes
Rosiek and Batlles, 2008 [10]	GSM/GPRS modem	External EEPROM	yes
Koutroulious *et al.*, 2003 [11]	TTL/RS-232 transceiver	PC hard disk	no
Lundqvist *et al.*, 1997 [12]	TTL/RS-232 transceiver	PC hard disk	no
GTZ, 1991 [13]	TTL/RS-232 transceiver	CMOS-RAM	no
